# Editorial: New ideas in: psychology for clinical settings 2022

**DOI:** 10.3389/fpsyg.2024.1356293

**Published:** 2024-03-13

**Authors:** Giulia Landi, Burkhard Brosig, Eliana Tossani

**Affiliations:** ^1^Department of Psychology “Renzo Canestrari”, University of Bologna, Bologna, Italy; ^2^Laboratory of Psychosomatics and Clinimetrics, Department of Psychology, University of Bologna, Cesena, Italy; ^3^Family Psychosomatics, Department of Pediatrics and Neonatology, Justus-Liebig-University Giessen, Giessen, Germany

**Keywords:** editorial, individualized treatment, mental health, innovation, clinical psychology

In the realm of clinical psychology, the quest for innovative and effective approaches is unending. The Research Topic “*New ideas in psychology for clinical settings 2022*” of *Frontiers in Psychology* stands as a testament to this ongoing journey. The papers on this Research Topic not only illuminate diverse aspects of psychological research and practice but also converge on a pivotal theme: the need for a more individualized, context-specific, and systemic approach to mental health care. In this editorial, we delve into each contribution, unraveling how they collectively chart a course toward a more nuanced understanding and treatment of psychological disorders.

## Individualized approaches in mental health

The manuscripts by Ong et al. and Cowden et al. highlight a significant shift in clinical psychology—toward more personalized and adaptable treatment methods. Ong et al.'s exploration of Process-Based Therapy (PBT) marks a significant evolution in cognitive-behavioral therapy. By incorporating principles of evolution science and idiographic methods, PBT offers a more flexible framework that can be customized to the unique psychological processes of individuals. This approach embodies the shift toward patient-centered models, acknowledging the diversity and complexity of human experiences in psychological disorders. In their manuscript, Ong et al.'s illustrate a case study demonstrating the application of PBT tools and principles. They focus on delivering treatment that is both informed by the process and centered around the individual, offering insights and recommendations for effectively implementing PBT in clinical settings. Similarly, Cowden et al.'s investigation into the distinction between depression and suffering unravels the intricate layers of psychological distress. The findings of their cross-sectional study emphasize the importance of discerning various facets of psychological distress, advocating for screening and addressing suffering as a form of distress that is distinct from depression. This nuanced understanding is crucial for clinicians, as it enables them to tailor interventions more precisely to individual patient needs.

Continuing with this theme of individualized care, Åkerlund et al. explored gender-specific auditory processing differences in Autism Spectrum Disorders (ASD). They found that females with ASD showed an auditory advantage in unisensory processing, leading to fewer social communication issues, while multisensory processing led to more problems in social awareness. In males, a visual dominance correlated with increased social rigidity issues. The study suggests that the female advantage in unisensory processing could explain the higher prevalence of ASD in males. These findings are a call to action for clinicians and researchers alike to adopt gender-sensitive approaches in understanding and treating ASD, recognizing the diversity within the spectrum.

## Systemic factors and trauma-informed approaches in clinical settings

The studies by Zeng et al. and Nikopaschos et al. turn the spotlight on the broader systemic factors influencing mental health outcomes. Zeng et al. examine the impact of inclusive leadership on reducing turnover intentions among Chinese nurses during the COVID-19 pandemic. Findings from their large cross-sectional multicenter study reveal how organizational culture and leadership styles play a pivotal role in the mental wellbeing of healthcare professionals. This research underscores the need for fostering supportive and inclusive work environments in healthcare settings, which can mitigate psychological distress and enhance job satisfaction.

Nikopaschos et al.'s conducted a study on Trauma-Informed Care (TIC) in an adult acute inpatient mental health unit, demonstrating its effectiveness. Their research, spanning 4 years, showed that implementing two trauma-informed practices—Power Threat Meaning Framework Team Formulation and Psychological Stabilization training—significantly reduced self-harm, seclusion, and restraint use in this clinical setting. The model of TIC underscores the necessity of trauma-informed care not only in acute inpatient mental health settings but also in crisis and community mental health services. It advocates for a more compassionate and thorough approach to treating individuals in these contexts, emphasizing the need for care that is informed by an understanding of trauma.

## Addressing the needs of special populations

Botschek et al. and Möhring et al. bring to light the importance of addressing the specific needs of diverse populations. Botschek et al.'s evaluated two multidimensional pediatric-psychosomatic inpatient treatments in Germany for disorders like pediatric dissociative, mood, and somatoform, along with chronic somatic conditions. Both treatments integrated individual and family therapy, along with group, art, music, and physiotherapy, but differed in approach: clinic A integrated psychodynamic and behavioral methods, while Clinic B focused on psychoanalysis and family dynamics. Results showed improvement in internalizing problems in both, but only Clinic A saw significant alexithymia reduction. Nevertheless, taking into account treatment duration, these differences became statistically insignificant. Results highlight that the duration of treatment might be as influential as the therapeutic approach itself. This finding brings to light the significance of time and continuity in therapeutic interventions, especially for young individuals navigating complex psychological issues.

Finally, Möhring et al. discuss an innovative counseling model for young adults from challenging backgrounds, particularly those who have dropped out of school. Their approach grounded in the theories of identity development and defense mechanisms as proposed by Vaillant and Erikson, effectively combines professional support with therapeutic environments. It demonstrates the effectiveness of adapting therapeutic environments to meet specific needs. This model, characterized by its low-threshold, destigmatizing, and flexible nature, offers a promising pathway to engage with and support marginalized or hard-to-reach populations effectively.

## Conclusion

The “*New ideas in psychology for clinical settings 2022*” issue collectively underscore the evolving landscape of clinical psychology. These studies pave the way for a more responsive, adaptable, and individualized approach to mental health care, emphasizing the need for patient-centered treatments, recognition of systemic factors, and the importance of trauma-informed care in various clinical settings. As the field of clinical psychology continues to evolve, the insights garnered from these studies will inform future research and practice, ensuring that psychological care becomes more inclusive, empathetic, and tailored to the diverse needs of individuals.

## Author contributions

GL: Writing – original draft, Writing – review & editing. BB: Writing – original draft, Writing – review & editing. ET: Writing – original draft, Writing – review & editing.

